# Fatigue presentation, severity, and related outcomes in a prospective cohort following post-COVID-19 hospitalization in British Columbia, Canada

**DOI:** 10.3389/fmed.2023.1179783

**Published:** 2023-06-29

**Authors:** Tianna Magel, Emily Meagher, Travis Boulter, Arianne Albert, Melody Tsai, Carola Muñoz, Chris Carlsten, James Johnston, Alyson W. Wong, Aditi Shah, Chris Ryerson, Rhonda Jane Mckay, Luis Nacul

**Affiliations:** ^1^Women’s Health Research Institute, British Columbia Women’s Hospital, Vancouver, BC, Canada; ^2^Division of Respiratory Medicine, Department of Medicine, University of British Columbia, Vancouver, BC, Canada; ^3^Centre for Heart Lung Innovation, St. Paul’s Hospital, Vancouver, BC, Canada; ^4^General Internal Medicine, Department of Medicine, University of British Columbia, Vancouver, BC, Canada; ^5^Department of Family Practice, Faculty of Medicine, University of British Columbia, Vancouver, BC, Canada; ^6^CureME, London School of Hygiene and Tropical Medicine, London, United Kingdom

**Keywords:** Long-COVID, post-COVID fatigue syndrome, post-COVID fatigue, chronic fatigue syndrome, myalgic encephalomyelitis

## Abstract

**Introduction:**

Increasing evidence on long-term health outcomes following SARS CoV-2 infection shows post-viral symptoms can persist for months. These symptoms are often consistent with those of Myalgic Encephalomyelitis or Chronic Fatigue Syndrome (ME/CFS). The aim of the present study was to examine the prevalence and outcome predictors of post-viral fatigue and related symptoms 3- and 6-months following symptom onset.

**Methods:**

A prospective cohort of patients hospitalized with Coronavirus disease (COVID-19) (*n* = 88) were recruited from a Post-COVID-19 Respiratory Clinic (PCRC) in Vancouver, Canada to examine predictors of long-term fatigue and substantial fatigue. Multivariable mixed effects analyses examined the relationship between patient predictors, including pre-existing comorbidities, patient reported outcome measures, and fatigue and substantial fatigue at follow-up.

**Results:**

The number of patients experiencing fatigue or substantial fatigue at 3 months post-infection were 58 (67%) and 14 (16%) respectively. At 6 months these numbers declined to 47 (60%) patients experiencing fatigue and 6 (6%) experiencing substantial fatigue. Adjusted analysis, for sex, age, and time, revealed the number of pre-existing comorbidities to be associated with fatigue (OR 2.21; 95% CI 1.09–4.49; 0.028) and substantial fatigue (OR 1.73; 95% CI 1.06–2.95; 0.033) at 3 months follow-up. Except for shortness of breath, self-care, and follow-up time, all follow-up variables were found to be associated with fatigue and substantial fatigue at 3 months.

**Conclusion:**

Fatigue and substantial fatigue are common after COVID-19 infection but often diminish over time. A significant number of patients continue to exhibit long-term fatigue at 6 months follow-up. Further research is needed to clarify the causality of viral infections in the development and severity of fatigue as a symptom and in meeting post-viral fatigue syndrome or ME/CFS diagnostic criteria.

## 1. Introduction

Rapid global spread of Coronavirus disease (COVID-19) has resulted in an estimated 757 million confirmed cases and approximately 6.8 million deaths worldwide as of February 2023 (1). There have been extensive investigations into acute stages of viral infection however, less is known about the long-term impacts experienced after infection. As the number of patients who have recovered from COVID-19 grows, it is evident that “recovery” is not synonymous with a return to previous health status for many individuals.

Emerging evidence demonstrates that, for a significant number of individuals, post-COVID-19 sequalae persists well beyond the acute stages of viral infection (2–5). Estimates for the proportion of people who experience post-COVID conditions vary. However, symptoms that persist for more than 12 weeks are termed “Long-COVID’ or Post-Acute Sequalae of coronavirus 2 (SARS-CoV-2) (6, 7). The Center for Disease Control estimates that over 30% of hospitalized individuals experience post-COVID related symptoms for 6 months or longer after infection (8).

COVID-19 is now recognized as a systemic disease with multiorgan involvement (9). Fatigue and cognitive impairment, along with abnormal respiratory function and other enduring neuropsychiatric and physical manifestations have been reported as the most prevalent and debilitating symptoms of post-COVID conditions (3, 10, 11). Literature examining long-term outcomes shows, for those hospitalized, post-COVID-19 symptoms can persist for upwards of 12 months (4). Indeed, many may experience long-term symptoms congruent with that of Myalgic Encephalomyelitis/Chronic Fatigue Syndrome (ME/CFS), such as persistent or debilitating fatigue, also referred to as post-COVID-19 fatigue syndrome (3, 7).

Fatigue and other long-COVID related outcomes are varied across studies due to poor standardization of data collection methods and measurement tools (12). Standardized investigations into the presentation of fatigue and related symptoms after viral infection is critical to providing evidence-based post-viral care for those experiencing severe long-term forms of COVID-19 infection. In this study, we examine the clinical presentation of post-viral fatigue within a prospective cohort of individuals hospitalized with severe COVID-19 in Vancouver, Canada. Hospitalization and follow-up predictors of fatigue and patient-reported clinical outcomes are described at 3- and 6-months post-symptom onset of SARS-CoV-2 infection.

## 2. Materials and methods

### 2.1. Measurements

This study involved a prospective cohort of 88 adult individuals (≥18 years) recruited from the Post-COVID-19 Recovery Clinic (PCRC) in Vancouver, Canada. Participants included those with a confirmed SARS-CoV-2 infection who were hospitalized from March to June 2020. Detailed methods involving this cohort as well as 3- and 6-month respiratory outcomes, and some patient-reported outcome measures (PROMs) have been previously reported elsewhere (13, 14). Briefly, at hospitalization (admission) and 3- and 6-months post-symptom onset, patient medical history, clinical variables, and patient-completed standardized questionnaires were collected. Clinical predictors of fatigue included age, sex, number of pre-existing comorbidities, Intensive Care Unit (ICU) admission, and mechanical ventilation. Pre-existing comorbidities were characterized as any pre-existing lung, cardiac, liver, cerebrovascular, renal, or autoimmune disease, diabetes, Gastroesophageal reflux disease (GERD), blood clots, and/or malignancy. Patient-reported predictors included scoring on the EuroQoL 5-Dimensions (EQ-5D), the Frailty Index (FI), the University of California San Diego Shortness of Breath Questionnaire (UCSD), Patient Health Questionnaire-9 (PHQ-9), and the Pittsburgh Sleep Quality Index (PSQI).

The EQ-5D is a five dimension preference-based and quality-of-life assessment. Scores from the EQ-5D were converted to a health utility index whereby scores of 1 represent perfect health and scoring of 0 is death (15). The UCSD was used to assess the severity and impact of dyspnea (shortness of breath) on daily activities, with scores greater than 10 reflecting dyspnea (16, 17). PHQ-9 scores assessed the severity of depression symptoms through 9 questions, with higher scores indicating higher severity (18). Assessments of sleep quality and sleep patterns were ascertained using the PSQI which consists of seven domains (19). This analysis only considered the PSQI global score which is a summation of all sleep-related domains. Finally, aging and vulnerability to adverse outcomes were determined using the FI, which is scored from 0 to 1, with higher scores indicating greater frailty (20). FI is calculated as the proportion of deficits present out of a list of 40 potential health deficits across multiple organ systems.

### 2.2. Outcomes

Primary outcomes included the presentation and severity of persistent fatigue at 3- and 6-months post-symptom onset. Fatigue was classified as an individual reporting feeling tired or having little energy for several days or more over the last 2 weeks (PHQ-9) as well as, indicating “always feeling tired” (FI). Substantial fatigue was classified as an individual reporting fatigue, as defined above, and “slight” differences in their ability to conduct usual activities (EQ-5D).

### 2.3. Statistical analyses

Descriptive statistics were used to describe participant characteristics at the time of hospitalization. Associations between all predictors (i.e., clinical and PROMs) and presentation of fatigue and substantial fatigue at 3- and 6-months were examined using multivariable mixed effect logistic regression modeling to account for correlations between timepoints and bivariate analyses were used to determine potential discrepancies between those with and without fatigue at each timepoint. Adjusted analysis of all predictor variables at 3- and 6-month timepoints did not include the EQ-5Ds “self-care” dimension due to a lack of variance in responses. Given the low number of patients with substantial fatigue at 6 months follow-up (*n* = 6), modeling for substantial fatigue included only the 3-month follow-up period. McNemar’s test was then used to examine changes in the presence of substantial fatigue across time. Relationships between clinical predictors and PROMs were examined using multivariable mixed effects linear regression modeling. Statistical significance was determined by a two-sided *p*-value of <0.05. All analyses were conducted using the statistical software package R (Version 4.2.1).

## 3. Results

Table 1 shows the main characteristics of the cohort at hospitalization and 3- and 6-months as well as the prevalence of fatigue. Among those included in this analysis (*n* = 88), mean age was 61.1 (±16.2) years, 63.6% were male, 45.5% identified as white, 80.7 and 89.8% had no pre-existing lung or autoimmune disease, respectively. Approximately 48.2% were admitted to ICU, of whom 20.5% were on mechanical ventilation during hospitalization. Prevalence of fatigue and substantial fatigue were reported to be 66.7% and 16.1%, respectively, at 3 months. By 6-month follow-up, fatigue was exhibited in 59.5% and substantial fatigue in 6.9% of patients. Among participants with and without fatigue at 3- and 6- months, bivariate analysis indicated statistically significant differences in participant reported scoring on PHQ-9, EQ-5D, shortness of breath, and overall health scoring (Table 2).

**Table 1:** Characteristics of PCRC* cohort and presence of Fatigue at 3- and 6-months.

Variables	All PCRC participants (*N* = 88)
Age	61.1 (±16.2)
Sex	
Male	56 (63.6)
Female	32 (36.4)
Ethnicity	
Asian	35 (39.8)
Other	13 (14.8)
White	40 (45.5)
Pre-existing comorbidities	1.5 (±1.3)
Lung disease	17 (19.3)
Cardiac disease	46 (52.3)
Diabetes	20 (22.7)
Cerebrovascular disease	4 (4.5)
Gastroesophageal reflux disease	12 (13.6)
Liver disease	4 (4.5)
Renal disease	10 (11.4)
Autoimmune disease	9 (10.2)
Blood clots	1 (1.1)
Malignancy	8 (9.1)
ICU*	41 (48.2)
Mechanical ventilation*	17 (20.5)
Fatigue	
3 Months	58 (66.7)
6 Months	47 (59.5)
Substantial fatigue	
3 Months	14 (16.1)
6 Months	6 (6.9)

*Post-COVID-19 Respiratory Clinic (PCRC); Data shown are mean ± standard deviation or number (%).

*Missing information for 3 individuals for ICU and 5 individuals for mechanical ventilation.

**Table 2:** Characteristics of fatigue and no fatigue among participants at 3- and 6-months follow-up.

	Fatigue 3 months				Fatigue 6 months			
	Total	No	Yes	*p*-value	Total	No	Yes	*p*-value
	*N* = 87	*N* = 29	*N* = 58		*N* = 79	*N* = 32	*N* = 47	
Age; Mean (SD)	61.4 (16.1)	65.1 (11.7)	59.5 (17.7)	0.12	60.9 (16.6)	62.5 (14.8)	59.8 (17.9)	0.47
Sex								
Male; n (%)	55 (63.2%)	20 (69%)	35 (60.3%)	0.49	49 (62%)	20 (62.5%)	29 (61.7%)	1.0
Female; n (%)	32 (36.8%)	9 (31%)	23 (39.7%)		30 (38%)	12 (37.5%)	18 (38.3%)	
Ethnicity								
Asian; n (%)	34 (39.1%)	10 (34.5%)	24 (41.4%)	0.25	33 (41.8%)	15 (46.9%)	18 (38.3%)	0.33
White; n (%)	40 (46%)	12 (41.4%)	28 (48.3%)		37 (46.8%)	12 (37.5%)	25 (53.2%)	
Other; n (%)	13 (14.9%)	7 (24.1%)	6 (10.3%)		9 (11.4%)	5 (15.6%)	18 (38.3%)	
Number Pre-existing comorbidities; Mean (SD)	1.5 (1.3)	1.1 (1.1)	1.7 (1.4)	0.060	1.5 (1.3)	1.3 (1.3)	1.6 (1.3)	0.36
ICU								
No; n (%)	44 (50.6%)	15 (51.7%)	29 (50%)	1.0	39 (49.4%)	14 (43.8%)	25 (53.2%)	0.81
Yes; n (%)	41 (47.1%)	14 (48.3%)	27 (46.6%)		37 (46.8%)	15 (46.9%)	22 (46.8%)	
Missing	2 (2.3%)	0 (0.0%)	0 (0.0%)		3 (3.8%)	3 (9.4%)	0 (0.0%)	
Mechanical ventilation								
No; n (%)	66 (75.9%)	21 (72.4%)	45 (77.6%)	0.57	61 (77.2%)	22 (68.8%)	39 (83%)	0.54
Yes; n (%)	17 (19.5%)	7 (24.1%)	10 (17.2%)		13 (16.5%)	6 (18.8%)	7 (14.9%)	
Missing; n (%)	4 (4.6%)	1 (3.4%)	3 (5.2%)		5 (6.3%)	4 (12.5%)	1 (2.1%)	
PHQ-9 score^a^; Mean (SD)	3.6 (4.8)	0.3 (0.8)	5.8 (5.2)	<0.0001	3.6 (4.8)	0.3 (0.8)	5.8 (5.2)	<0.0001
Shortness of breath score^b^; Mean (SD)	17.2 (19.8)	7.4 (13.6)	22.1 (20.7)	0.0009	15.8 (18.0)	6.1 (8.6)	22.4 (19.8)	<0.0001
EQ-5D score^c^; Mean (SD)	7.9 (3.6)	5.5 (0.9)	9.1 (3.8)	<0.0001	7.5 (2.9)	5.8 (3.2)	8.8 (3.5)	<0.0001
Missing	3 (3.4%)	2 (6.9%)	1 (1.7%)		–	–	–	
Health today (0–100); Mean (SD)	75.8 (15.5)	87.4 (9.4)	70.1 (14.7)	<0.0001	80.7 (12.5)	87.5 (9.2)	76.0 (12.4)	<0.0001

^a^Patient Health Questionnaire-9 (PHQ-9 Score), scores range from 0 to 27; 0 = None-minimal and 27 = Severe.

^b^Shortness of Breath (UCSD), scores range 0 to 120; high scores indicate greater dyspnea.

^c^EuroQoL 5-Dimensions (EQ-5 Score), scores range from 0 to 1; 0 = Death and 1 = Perfect Health; “Self-care” not modeled due to lack of variance.

### 3.1. Hospitalization predictors of fatigue at 3 and 6 months

**Figure 1:**
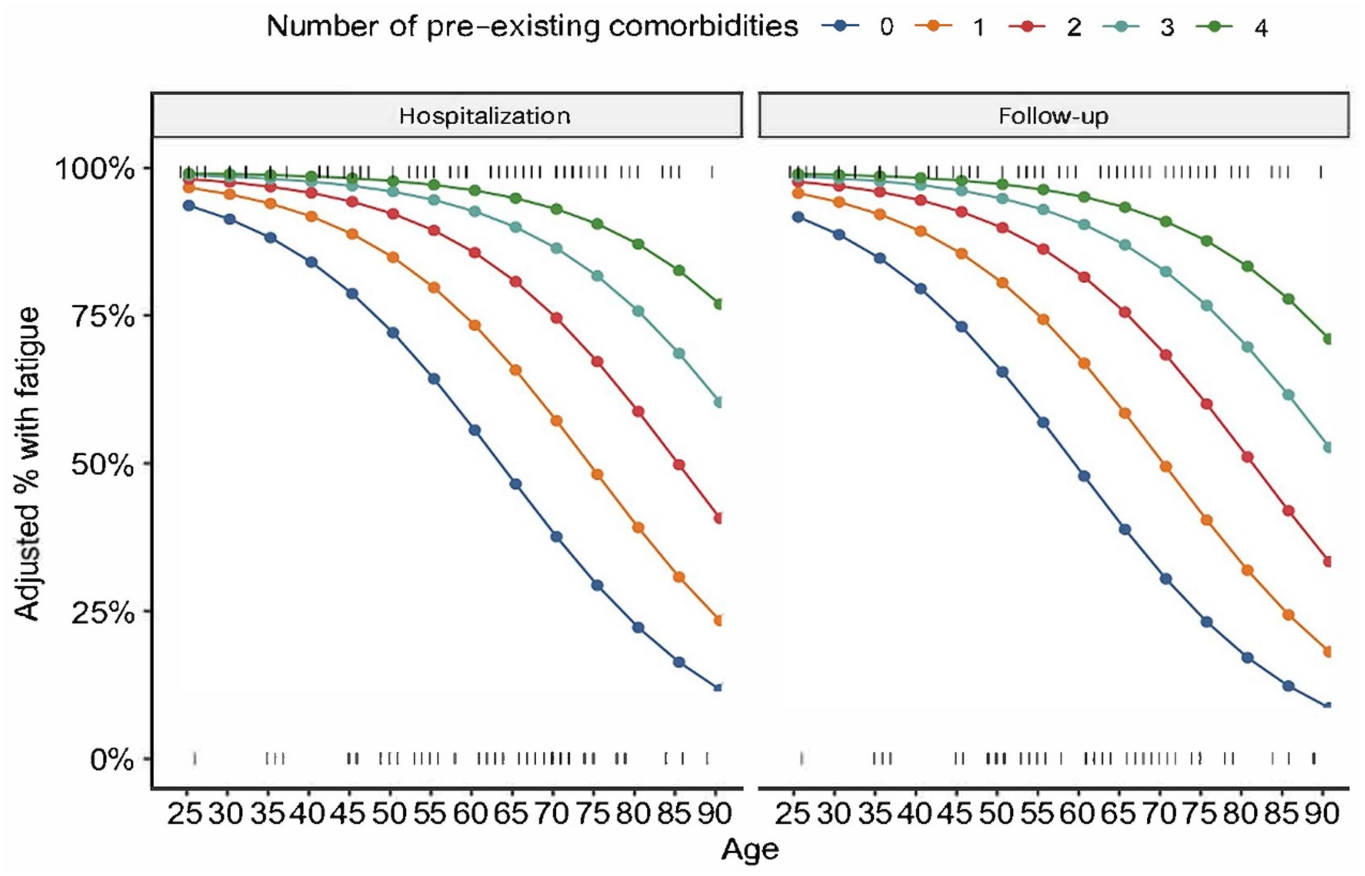
Fatigue and number of comorbidities at hospitalization and follow-up. The present figure shows the relationship between age, comorbidities and proportion with fatigue controlling for other variables in the multivariable model (no difference in proportions at 3 and 6 months).

The number of pre-existing comorbidities had a trend toward association with fatigue at 3 months (*p* = 0.06). Adjusted multivariable analysis, controlling for age, sex, and time at the point of hospitalization, revealed number of comorbidities (OR 2.21; 95% CI 1.09–4.49; *p* = 0.028) to be a predictor of fatigue at 3 months post-viral infection (Table 3 and Figure 1). Interestingly, age demonstrated a subtle protective effect in the likelihood of developing fatigue by 3 months follow-up (OR 0.93; 95% CI 0.88–0.98; *p* = 0.012). Patients who did not exhibit fatigue at 3 months did not go on to exhibit fatigue at 6 months follow-up.

**Table 3:** Adjusted multivariate model of fatigue at hospitalization.

Predictors	Fatigue		
	OR	CI	*p*-value
Time (follow-up)	0.73	0.29–1.83	0.505
Sex			
Male	Ref	–	–
Female	1.32	0.31–5.59	0.707
Age	0.93	0.88–0.98	**0.012**
Number of comorbidities	2.21	1.09–4.49	**0.028**
ICU	1.47	0.29–7.46	0.645
Mechanical ventilation			
No	Ref	–	–
Yes	0.29	0.03–2.44	0.256

Bolded values indicate *p* ≤0.05.

### 3.2. Predictors of fatigue at follow-up

Adjusted analysis, controlling for age and number of comorbidities, revealed correlations between fatigue and all variables measured at 3- and 6-months, with the exception of self-care and follow-up time (Table 4).

**Table 4:** Mixed effect modeling of PROMs^*^ follow-up variables and fatigue, adjusted for age, time, and number of pre-existing comorbidities.

	Fatigue		
	OR	95% CI	*p*
PHQ-9 score^a^	5.05	1.92–13.31	0.001
EQ-5D score^b^	2.35	1.65–3.36	<0.001
Usual activities	11.19	2.83–44.22	0.001
Mobility	5.39	1.96–14.82	0.001
Anxiety/depression	6.08	2.43–15.26	<0.001
Pain/discomfort	4.00	1.86–8.61	<0.001
Shortness of breath score^c^	1.09	1.04–1.14	<0.001
PSQI score^d^	1.71	1.24–2.36	0.001

^*^Patient-Reported Outcome Measures (PROMs).

^a^Patient Health Questionnaire-9 (PHQ-9 Score), scores range from 0–27; 0 = None-minimal and 27 = Severe.

^b^EuroQoL 5-Dimensions (EQ-5 Score), scores range from 0 to 1; 0 = Death and 1 = Perfect Health; “Self-care” not modeled due to lack of variance.

^c^Shortness of Breath (UCSD), scores range 0–120; high scores indicate greater dyspnea.

^d^Pittsburgh Sleep Quality Index (PSQI Score); scores range 0–21, higher scores indicate worse sleep quality.

### 3.3. Substantial fatigue: hospitalization variables

Fourteen individuals exhibited substantial fatigue at 3 months and six continued to exhibit substantial fatigue at 6 months follow-up. No patients developed new substantial fatigue between follow-up time periods. Adjusting for age, the number of pre-existing comorbidities at hospitalization was associated with substantial fatigue at 3 months follow-up (OR 1.73; 95% CI 1.06–2.95; *p* = 0.033, Table 5). No other hospitalization variables were associated with substantial fatigue.

### 3.4. Substantial fatigue: follow-up

With the exception of shortness of breath, all follow-up variables were associated with substantial fatigue at 3 months. Due to the small number of cases at 6 months, we did not examine this relationship further.

**Table 5:** Predictors of substantial fatigue at 3 months.

Hospitalization variables	Substantial fatigue		
	OR	95% CI	*p*
Sex (Female)	1.36	0.41–4.33	0.608
Age	0.74	0.39–1.44	0.358
Number of pre-existing comorbidities^a^	1.73	1.06–2.95	0.033
ICU admission	0.77	0.23–2.44	0.660
Ventilation	0.28	0.01–1.60	0.240
3 Month follow-up variables	OR	95% CI	*p*
PHQ-9^b^	1.43	1.23–1.78	<0.001
EQ-5D^c^	1.64	1.32–2.20	<0.001
Mobility	2.43	1.47–4.33	0.001
Usual activities	5.46	2.69–13.45	<0.001
Pain/discomfort	3.98	2.06–8.81	<0.001
Anxiety/depression	3.70	1.96–8.04	<0.001
Health today	0.92	0.87–0.96	<0.001
Shortness of breath	1.02	1.00–1.05	0.062
PSQI^d^	1.43	1.21–1.77	<0.001

^a^Pre-existing comorbidity includes any pre-existing lung, cardiac, liver, cerebrovascular, renal, or autoimmune disease, diabetes, GERD, blood clots, and/or malignancy; ^b^Patient Health Questionnaire-9 (PHQ-9 Score); ^c^EuroQoL 5-Dimensions (EQ-5 Score); ^d^Pittsburgh Sleep Quality Index (PSQI Score).

## 4. Discussion

The present study shows that long-term fatigue can persist for at least 6 months in 59.5% of patients previously hospitalized with SARS-CoV-2 infection. Within the PCRC cohort, reductions in the prevalence of fatigue and substantial fatigue were observed between 3- and 6-months follow-up. Greater improvements were observed among those experiencing substantial fatigue, which decreased to less than half of the original prevalence by the 6-month follow-up timepoint. These findings align with existing literature reporting a decreasing trend in the proportion of individuals experiencing fatigue across weeks since acute presentation (12, 21). Patients with more pre-existing comorbidities at the time of hospitalization were also found to be more likely to exhibit fatigue at 3- and 6- months post-viral infection. Patients who did not exhibit fatigue or substantial fatigue at 3 months did not go on to exhibit these symptoms at 6 months follow-up.

While contrary to some findings, an unexpected and subtle protective effect of age on the presence of fatigue was observed at follow-up timepoints. This finding is consistent with Subramanian et al. (22) who found that after adjusting for baseline covariates, age above 30 years was associated with a lower risk of reporting post-COVID symptoms. While protective effects against fatigue among those greater than 65 years of age has also been observed, (23) other literature indicates that rates of long-COVID increase with age from about 1–2% for those in their twenties, to about 5% among those in their sixties (24). Indeed, post-viral fatigue syndrome or ME/CFS may affect young people (<30 years) more often (25). These discrepancies may be attributed to differential reporting of symptoms according to age and other factors (e.g., those who are younger may be less accepting of feelings of disabling fatigue not previously experienced), and should be considered in future interpretations of fatigue and patient age. Additionally, those with very severe disease presentation on admission may have had lower expectations, in relation to being back to full health in the short-term, as compared to those with less severe SARS-CoV-2 infection. Research elucidating the role age and expectation of recovery play in long-term post-viral fatigue presentation necessitates further exploration.

Multimorbidity has also previously shown associations with post-viral fatigue syndrome symptoms (3, 22). Our research revealed, controlling for age, sex, and time, that the number of pre-existing comorbidities a patient had was significantly associated with fatigue and its respective severity at 3 months follow-up. Interestingly, adjusted analysis of our cohort revealed dyspnea to be associated with fatigue at 3- and 6-months but was found to only be marginally associated with substantial fatigue. These findings are likely the result of the small sample size of individuals found to be experiencing substantial fatigue at follow-up timepoints. Using the same cohort of patients, Shah et al. (13) found dyspnea to be the most common and persistent COVID-19 recovery symptom, with 42% experiencing dyspnea at 6 months follow-up. Unexplained dyspnea, (i.e., not related to abnormalities in lung function tests or imaging), was reported in 14 and 19% of cases at 3- and 6-months, respectively (13). Dyspnea in post-COVID cases has been suggested to result from multiple pathophysiological mechanisms, (26) and has also been reported in ME/CFS cases related to other causes (27). This symptom is also reported in dysautonomia, a common occurrence in ME/CFS (28).

### 4.1. Future directions

Our findings highlight the importance of further examinations into the role viral infections have in the presentation of long-term fatigue and related multimorbidities. While the number of individuals experiencing fatigue after viral infection is expected to decrease at follow-up timepoints, there are a subset of individuals for whom fatigue presentation and severity will persist. It is important that clinical teams remain attentive to monitoring patients for long-term fatigue after infection with SARS-CoV-2, encouraging patients to engage in practices that can aid in mitigating fatigue severity and lasting post-COVID symptoms. The considerable proportion of patients within our study continuing to exhibit symptoms of fatigue at 6 months highlights the need for further investigations into evidence-based practices that can meet the needs of those experiencing long-term fatigue after acute viral infection. Despite the change in severity with newer variants of SARS-CoV-2, it is key for provincial initiatives such as the Post-COVID-19 Interdisciplinary Clinical Care Network and national networks to support continuous data collection to assist longitudinal studies in Long COVID. Our findings add to the growing body of literature illuminating the role viral infections, such as COVID-19, may have in the development of long-term symptoms and persisting fatigue.

### 4.2. Limitations

This study has several limitations. Namely, our findings may be limited by the small participant sample size. As such, we were unable to examine correlates of substantial fatigue at 6 months due to limited size and subsequent lack of power. This prohibited examination of differences in the predictors of substantial fatigue at 3- and 6-months follow-up and prevented further investigation into those no longer exhibiting substantial fatigue at 6 months. Likewise, given our inability to ascertain whether criteria for ME/CFS or post-viral fatigue were met, substantial fatigue was used as a proxy. It is therefore likely that the prevalence of post-viral fatigue syndrome found in this study is substantially higher than rates adhering to strict clinical guidelines. Gaps remain in knowledge surrounding which comorbidities may predispose individuals to greater levels of long-term fatigue and its severity after hospitalization. Several studies have identified associations between self-reported measures and fatigue related outcomes, including reductions in quality of life and cognitive impairment (3, 29). However, there are limitations in such measures, highlighting the importance of objective outcome measures. We also note that the generalizability of study findings is limited to the sample of patients hospitalized with COVID-19 and cannot be extrapolated to those not exhibiting symptoms of COVID-19 or those who were not hospitalized.

## 5. Conclusion

After hospitalization, a significant proportion of individuals recovering from COVID-19 will continue to experience lingering symptoms for up to 6 months. For many patients, the presentation of post-viral symptoms will manifest in increased levels of fatigue. Further investigation into the presentation of fatigue, post-COVID infection, is needed to support evidence-based care and management for individuals experiencing long-term post-viral symptoms.

## Data availability statement

The raw data supporting the conclusions of this article will be made available by the authors, without undue reservation.

## Ethics statement

The studies involving human participants were reviewed and approved by the University of British Columbia Research Ethics Board. The patients/participants provided their written informed consent to participate in this study.

## Author contributions

AA performed the statistical analysis. TM and LN wrote the first draft of the manuscript. EM wrote a section of the manuscript. TB organized the database. All authors contributed to manuscript revision, read, and approved the submitted version.

## Conflict of interest

The authors declare that the research was conducted in the absence of any commercial or financial relationships that could be construed as a potential conflict of interest.

## Publisher’s note

All claims expressed in this article are solely those of the authors and do not necessarily represent those of their affiliated organizations, or those of the publisher, the editors and the reviewers. Any product that may be evaluated in this article, or claim that may be made by its manufacturer, is not guaranteed or endorsed by the publisher.
